# The Medical Directorship of the Exposition

**Published:** 1899-10

**Authors:** 


**A Monthly Review of Medicine and Surgery.**

*EDITOR:*

WILLIAM WARREN POTTER, M. D.

All communications, whether of a literary or business nature, books for review and exchanges should be addressed to the editor: 284 FRANKLIN STREET, BUFFALO, N. Y.

VOL. XXXIX.—LV. OCTOBER, 1899. No. 3.

### THE MEDICAL DIRECTORSHIP OF THE EXPOSITION.

THE proposition has been advanced by a newspaper that it is quite possible the Pan-American Executive Committee will find it difficult to appoint a medical director for the exposition. In support of this stand the newspaper in question pointed out that professional jealousies would seriously interfere with the naming of a man. Then the question of compensation was taken up, and quite unblushingly this newspaper declared that the honor of the position would in a large measure pay for the physician's time, that it was a responsible post and one which would bring its appointee prominently before the public.

All this may be true. The man who is appointed medical director will unquestionably be before the public. He will also practically be responsible for the health and welfare of the thousands upon thousands of people who will attend the exposition. There is a certain amount of honor connected with the position, but there is, too, a great responsibility, and the compensation should be adequate and generous.

The appointments which have been already made, carry with them good salaries and likewise considerable honor. There was not at any time any disposition shown to pay for the work to be done in honor, and there should not be in the case of the medical director.

Any physician who would accept the post at a niggardly—or, to be less harsh, let us say nominal—salary would be reflecting great discredit upon his profession and discounting his own ability.

The medical department of the coming exposition is the most important of all. How long, pray, would the exposition last, did an epidemic break out? The work of a medical director must be ceaseless, and he should be paid proportionately.

If the Pan-American Exposition executive committee has any idea of appointing as medical director a man who will accept the place at a niggardly—again, let us be less harsh and say nominal—salary, the JOURNAL would suggest that a funeral director and a superintendent of cemeteries be added to the working force, and that provision be made for a quarantine hospital.

The JOURNAL, however, does not believe the executive committee will permit itself to be influenced in any way by the newspaper referred to, but that it will, when the time comes, appoint as medical director, a man of wide experience, a man of standing and reputation, and, most important, a man of executive ability. The members of the committee are men of business experience and wisdom and would hardly err in so important a matter.

